# The Case of a 15-Year-Old With Non-Parasitic Chyluria

**DOI:** 10.7759/cureus.17388

**Published:** 2021-08-23

**Authors:** Tina Lovrec Krstić, Kristijan Šoštarič, Primož Caf, Matija Žerdin

**Affiliations:** 1 Radiology, University Medical Centre Maribor, Maribor, SVN

**Keywords:** chyluria, non-parasitic, ceus retrograde pyelography, mr lymphangiography, urinary retention

## Abstract

Chyluria is defined by the presence of chyle in urine, and is caused by a communication between the lymphatic and urinary system. Commonly, it is characterised by the excretion of milky white urine, which is present in up to 70% of chyluria cases. The prevalent cause for chyluria in Asia is filariasis with *Wuchereria bancrofti. *Non-parasitic chyluria is more common in western countries and is usually subsequent to traumatic factors, infections, or tumours. The occurrence of non-parasitic chyluria in the absence of trauma, iatrogenic or other, is exceedingly rare. The lymphatic system is rather challenging to visualize. Herein, we present a case of non-parasitic chyluria and our approach of combining different complementing imaging modalities, such as contrast-enhanced ultrasound (CEUS) retrograde pyelography and magnetic resonance (MR) lymphography.

## Introduction

Chyluria is a condition in which chyle is present in urine. It is endemic in certain parts of Asia (e.g., India), and Sub-Saharan Africa. Parasitic chyluria is 95% caused by *Wuchereria bancrofti* which is transmitted by Culex mosquitoes. While non-parasitic chyluria is rare, there are several possible causes: trauma, complication of surgery, infections, malignancy, lymphatic malformation, radiation, abscess, congenital abnormalities, stenosis of the thoracic duct, and others [1]. Chyluria typically presents with milky white urine (70% of cases), dysuria, urgency, urinary retention due to chylous clots, and sometimes even clot colics. Hematuria and hypoproteinaemia may be present. It can also be completely asymptomatic. Weight loss, malnutrition, chills, and peripheral edema have been noted amongst systemic manifestations [1-3]. In this study, we report a rare case of non-parasitic nontraumatic chyluria in a 15-year-old girl with an emphasis on imaging techniques.

## Case presentation

**Figure 1:**
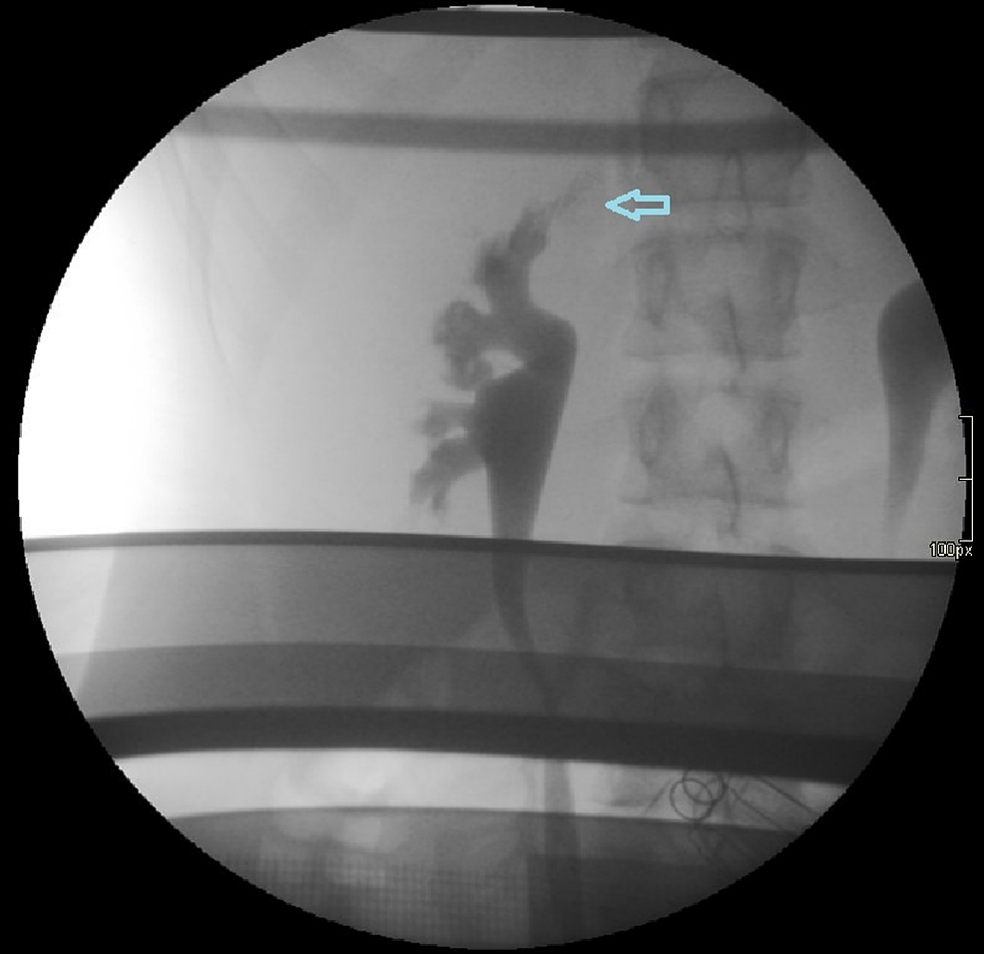
Fluoroscopic retrograde pyelography showing the point of leakage (arrow).

A 15-year-old girl with a near seven-year medical history of recurrent proteinuria, leukocyturia, hematuria, urinary tract infections, and vulvitis presented with difficulty to initiate urination and urinary obstruction that was often momentarily resolved by the excretion of mucus clots. The patient's urine was of characteristic milky appearance. The physical examinations found the patient to be in good overall condition. Laboratory findings demonstrated significant proteinuria and hematuria (Table 1). Chylomicrons and triglycerides were found in the patient’s urine indicating the presence of intestinal lymph. Although she had a few accompanying urinary tract infections, the urine was mostly found to be sterile. Laboratory results also showed vitamin D deficiency. Hypoproteinemia and hypoalbuminemia were detected. Ultrasound (US) examinations of the urinary tract revealed normal-sized kidneys with a bilateral partially extrarenal pyelon, slightly dilated on both sides. Voiding urosonography, uroflowmetry, cystometry, and vaginoscopy showed no abnormalities. Bilateral cystoscopy and fluoroscopic retrograde pyelography were performed, demonstrating a leak of contrast media into the right perirenal space, but the exact location of the leak could not be found (Figure 1).

**Table 1:** Laboratory values.

Exam	Value	Reference value
Urine proteins	10.28 g/24h	up to 0.15 g/24h
Urine erythrocytes	1165 cells/ μL	up to 17 cells/ μL
Urine triglycerides	2.7 mmol/L	up to 0,113 mmol/L
Serum vitamin D	26.1 nmol/L	47.7-144 nmol/L
Serum protein	48 g/L	57-80 g/L
Serum albumin	25 g/L	29-47 g/L

**Figure 2:**
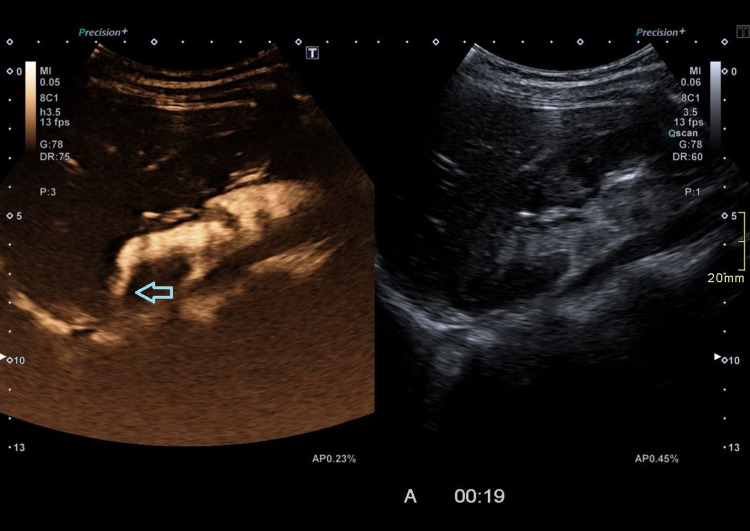
Contrast-enhanced ultrasound (CEUS) retrograde pyelography demonstrating the leak of contrast agent through the renal parenchyma (arrow).

**Figure 3:**
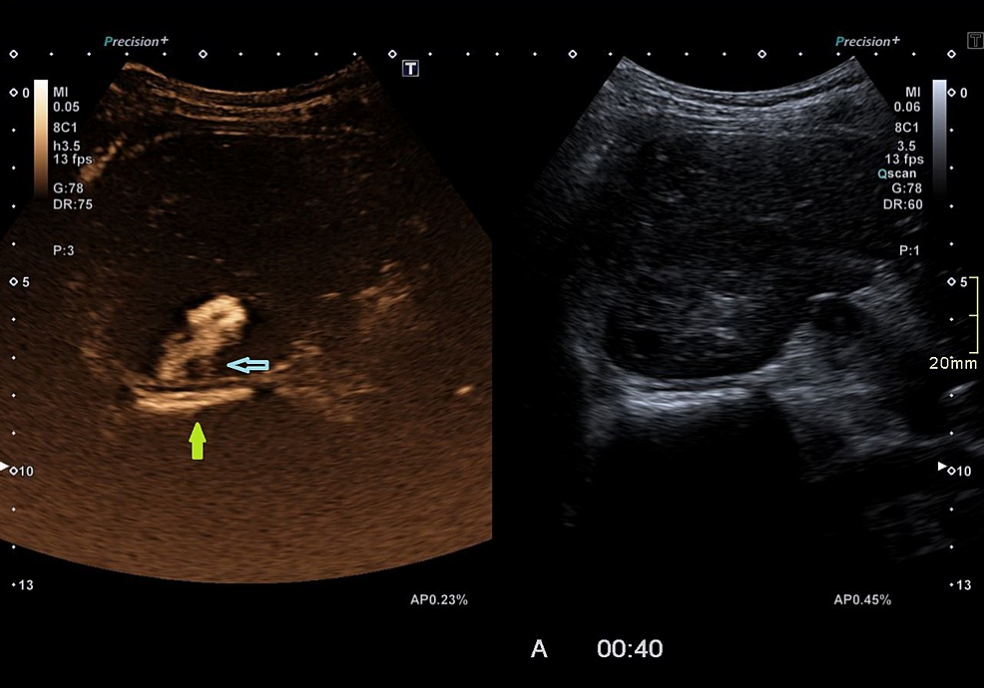
Contrast-enhanced ultrasound (CEUS) retrograde pyelography demonstrating the leak of contrast agent through the renal parenchyma (blue arrow) into the perirenal space (green arrow).

**Figure 4:**
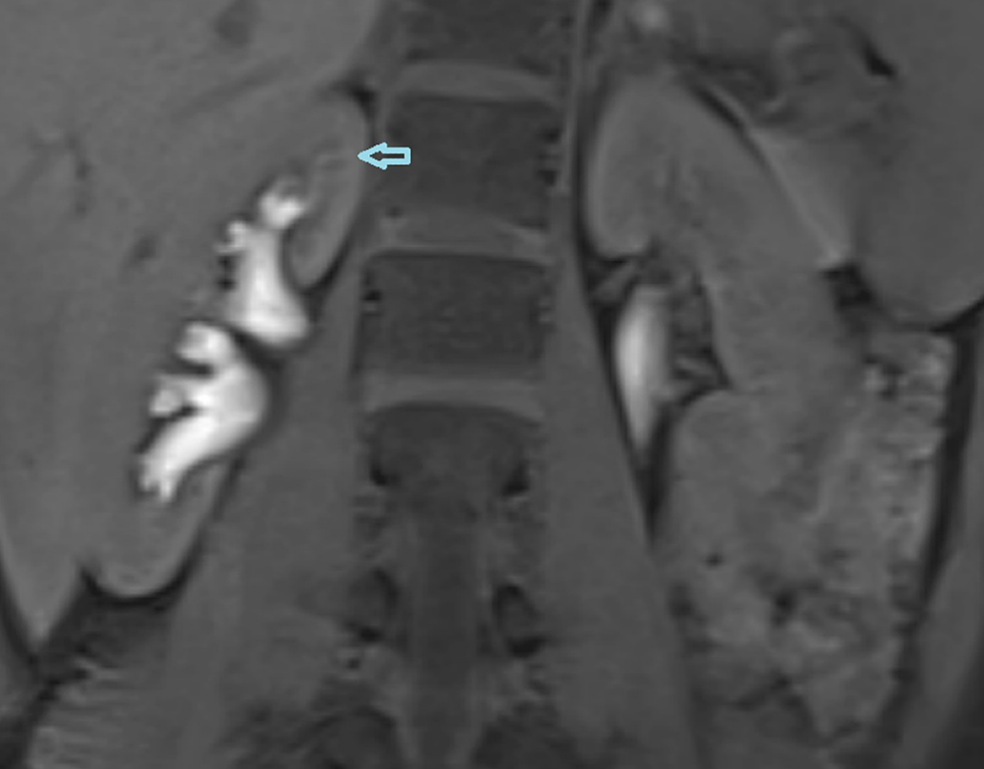
Magnetic resonance (MR) retrograde pyelography (VIBE FatSat T1 sequence) demonstrating the point of leakage. VIBE: volumetric interpolated breath-hold examination; FatSat: fat saturation.

**Figure 5:**
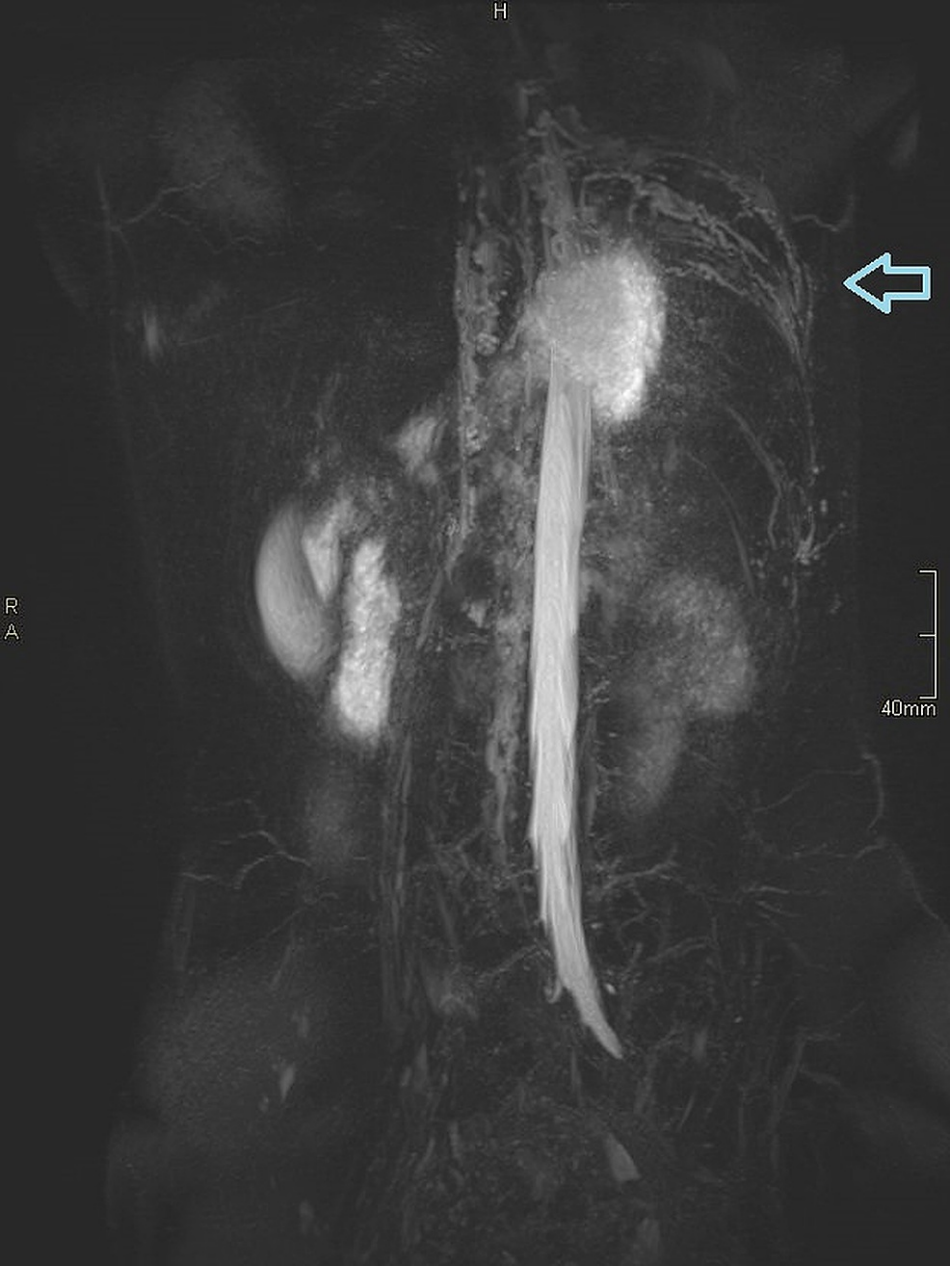
Magnetic resonance (MR) lymphography demonstrating the dilated and tortuous intercostal lymph vessels.

**Figure 6:**
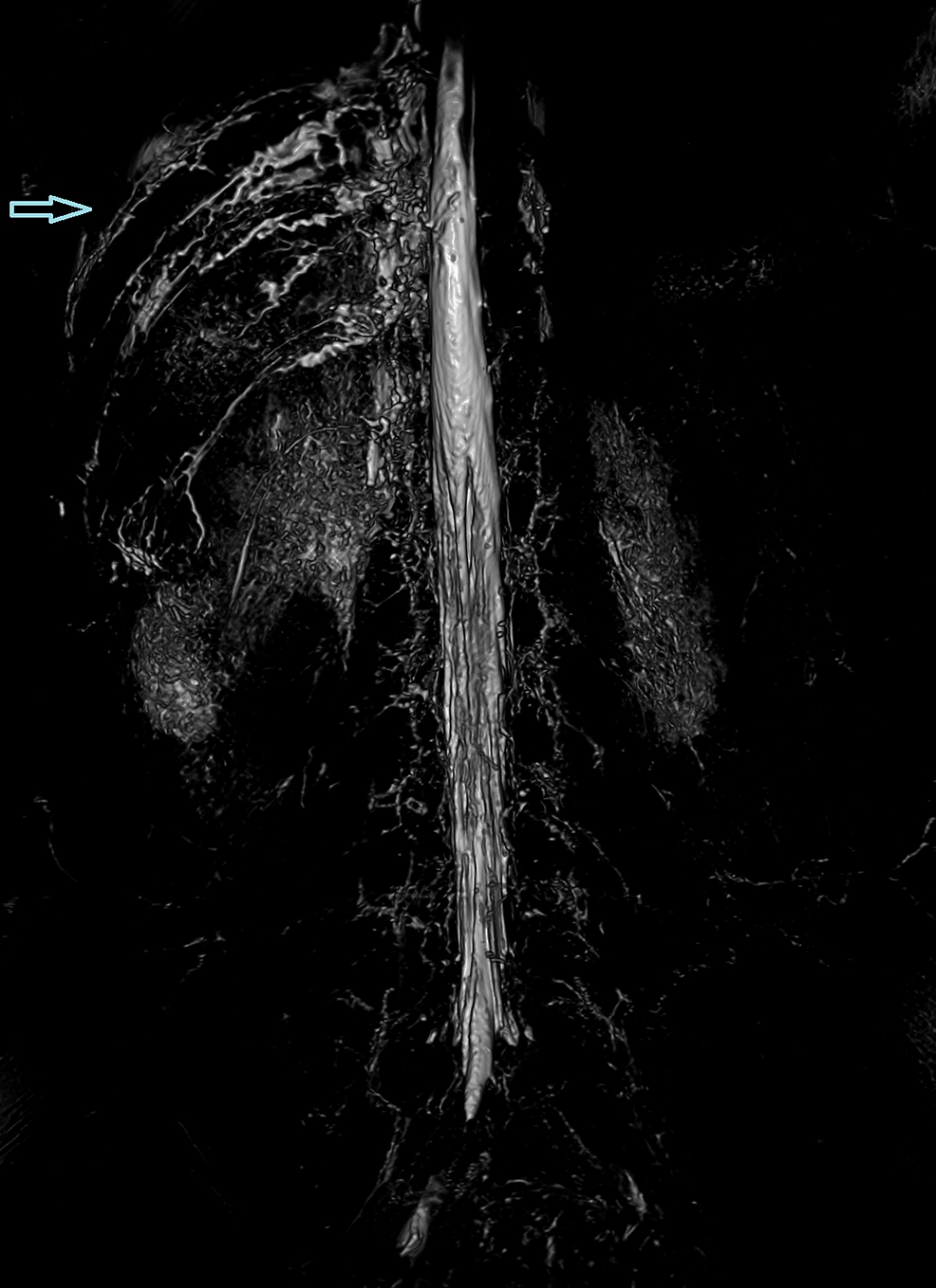
Magnetic resonance (MR) lymphography demonstrating the dilated and tortuous intercostal lymph vessels.

Therefore, contrast-enhanced ultrasound (CEUS) retrograde pyelography was performed with the application of US contrast through the previously inserted catheter. With this exam, a leak of contrast from the pyelon into the supero-posterior part of the right perirenal space was demonstrated, but no exact point of communication with the lymphatic system was established (Figures 2-3). Immediately after, the patient underwent an MR exam with a modified protocol for the imaging of the abdominal and thoracic lymph pathways. The sequences were as follows: T2 weighted sequence in the abdomen and thorax, then T2-weighted imaging (T2WI), T1 Dixon, diffusion-weighted imaging (DWI), and finally, after the application of gadolinium contrast, T1 Dixon. Gadolinium contrast was applied through the catheter in the right pyelon (Figure 4). The heavily T2WI sequences showed tortuous and ectatic lymphatic vessels in the abdominal paraaortic region - the left and right lumbar truncus. Collateral vessels in this area were also visualised. The right lumbar truncus ended intra-abdominally, entering the cisterna chyli, while the left was seen to extend up to the level of the 8th thoracic vertebra. An influx from the dilated and ectatic intercostal lymphatic vessels into the segment located between the 8th and 12th thoracic vertebra of the left lumbar truncus was observed (Figures 5-6). The cisterna chyli and thoracic duct showed no abnormalities. The point of junction between the thoracic duct and the venous system was not visualized due to artifacts caused by blood flow. On the native T2 weighted sequence, no convincing dilated lymphatic vessels were found in the immediate vicinity of the kidneys. The application of contrast in the MR exam failed to demonstrate the leak previously observed in the CEUS retrograde pyelography, presumably because the US contrast agent caused a temporary blockage of the pyelo-lymphatic fistula. We confirmed the lack of contrast leak on repeat CEUS retrograde pyelography right after the MR was performed. 

Three days later, another CEUS was performed, the leak was present again. Thus, we assume that the US contrast agent temporarily closed the communication between the lymphatic and urinary system. Possibly because the contrast bubbles are of relatively large size. One day later, an attempt was made to execute both MR pyelography and MR lymphangiography with ultrasound-assisted cannulation of the inguinal lymph nodes. The MR pyelography on this occasion demonstrated a slight leak that correlated with the one described previously in the CEUS examination. The MR lymphangiography was unsuccessful since the contrast extravasated due to the dislocation of the IV cannula, as the patient was moved from the bed to the MR table. 

The patient’s treatment mainly consisted of a diet restricting the intake of foods containing long-chain fats. To prevent malnutrition, the patient received supplements in the form of medium-chain triglyceride (MCT) oil. Due to deficiency, vitamin D was also supplemented. If the patient adhered to her diet, she experienced no discomforts in regards to urinary obstruction. An attempt was made to close the lypmho-urinary communication with a three-day application of 1% silver nitrate through the catheter in the right pyelon, with no notable success. A temporary closure of the communication was accidentally achieved during CEUS. The patient is awaiting a further attempt at MR lymphangiography, afterwards the possibility of treatment with surgical lympho-venous fistula will be discussed, depending on the result of lymphangiography.

## Discussion

The diagnosis of non-parasitic chyluria may be tricky as one must first consider it as a possible cause to begin the required diagnostic exams. Confirmation of chyluria may be done with a bedside chloroform or ether test that separates fat globules from the urine and forms an organic layer. Sudan III test may also be used. The most accurate method is the evaluation of triglyceride levels in urine [4,5]. In our case the levels of triglycerides were 2.7 mmol/L, we also found chylomicrons.

Imaging

Vizualisation of the lymphatic system in cases of chyluria is used to locate the site of leakage. Moreover, it can give insight into the anatomy of the lymphatic system, which is usually abnormal in patients with chyluria. In patients with chyluria dilated clusters of lymphatic channels are often found in the retropelvic, retroperitoneal and paraaortic regions [6]. Collateral, ectatic, and dilated blood vessels were also found in our patient. The lymphatic system has, due to the small size of the lymphatic vessels, proven to be quite challenging to visualize. Its size also presents an obstacle in contrast material application. T2 weighted MR imaging can be used to visualize lymphatic structures, it can demonstrate pathologic lymphatic structures, such as lymphatic masses, lymphangiectasias, and dilatations. Due to it being a static imaging modality, however, dynamic information is inadequate which curbs its use in cases of lymphatic reflux or leakage. It may also have difficulties visualizing smaller lymphatic ducts because of their poor signal. Overlapping fluid-containing structures can also impede visualization of the lymphatic ducts. Artifacts from breathing, peristalsis, and cardiac movement may also interfere. Dynamic contrast-enhanced MR lymphangiography was developed to overcome the disadvantages of T2 weighted MR imaging. It is performed by injecting a gadolinium-based contrast material (GBMC) into the groin lymph nodes under US control. In comparison to fluoroscopic lymphangiography, it is more sensitive in the detection of leakages, as GBMC is less viscous. One of its drawbacks is its inability to image lymphatic structures outside of the injected contrast agent’s route. Occasionally, an obstruction or leak prevents the propagation of contrast material into the thoracic duct. Another limitation is that the cannulation of lymph nodes is a very sensitive procedure, and even small movements may cause dislodgement of the cannula, leading to extravasation of the contrast. The studies we reviewed on this topic all reported a successful execution of contrast-enhanced MR lymphangiography in pediatric patients as well as adults, and even lead to changes in the treatment approach of some cases [7-9]. For the ideal visualization of the central lymphatic system, a combination of T2 weighted MR and contrast-enhanced MR lymphangiography is recommended [10].

Another possible imaging technique is lymphoscintigraphy using 99 m Tc-nano colloid. It is non-invasive, safe, and simple. It provides dynamic information, but its spatial resolution and anatomic details are lacking [10]. Using rigid cystoscopy to demonstrate chyle efflux from the ureteric orifice helps determine which side the chyluria originates from. It is also helpful in excluding other pathologies [4]. Fluoroscopy used to visualise the central lymphatic pathways may be performed with US-guided injection of oil-based contrast material. The disadvantages of this method include exposure to ionizing radiation, longer examination time, inadequate dynamic information, and insufficient information about the relationship of the lymphatic system with other structures and systems. Caution is also needed in children with right to left shunts, as the oil-based contrast might cause a paradoxical embolism. To demonstrate the communication between the lymphatic and urinary system, another X-ray exam may be used - conventional retrograde pyelography. Its drawbacks are similar to conventional lymphography. Although the contrast agent is not injected into the bloodstream, life-threatening anaphylactic reactions are still a possible complication if the contrast media is absorbed through the urothelium. Therefore, it should not be performed in patients with known contrast media allergies [11]. To avoid the use of ionic contrast media and radiation exposure, while also offering a simultaneous detailed intra-abdominal evaluation, MR retrograde pyelography may be used. Despite its advantages, its use is limited by the high cost and limited availability [11].

CEUS may serve as an excellent substitute for fluoroscopic retrograde pyelography. In our case, selective CEUS retrograde pyelography demonstrated the site of leakage in the kidney with comparable results to conventional fluoroscopic retrograde pyelography. Daneshi et al. have also studied and confirmed CEUS to be a viable alternative to fluoroscopic imaging of the urinary tract when determining the location of nephrostomas [12]. When applying this method, we observed that the US contrast agent may temporarily block the communication. We have not discovered anything similar being reported in other cases of chyluria, likely because CEUS was not utilised in such a way. It can also be used to confirm the needle position, before performing MR lymphangiography on any MR machine with a detachable table [13]. 

Treatment

The conservative approach to treat chyluria includes a high protein diet that minimizes the intake of triglycerides, especially the long-chain type. This decreases the production of chylomicrons, which would be transported via lymph, and helps reduce chyluria. Medium-chain triglycerides are directly absorbed into the bloodstream; therefore, they do not contribute to chyluria. Thus, foods like coconut milk and others that contain a high amount of the medium-chain variety are a safe option to prevent malnutrition, while adhering to this diet [14]. MCT oil is also a viable option as presented in this case. A treatment option for chyluria is endoscopic sclerotherapy. To induce chemical lymphangitis and edema, which leads to fibrosis, sclerosant agents, such as 0.1%-1% silver nitrate, 0.2%-5% povidone-iodine, 50% dextrose, 3% hypertonic saline, and contrast media, are instilled endoscopically into the ureter. Silver nitrate is effective in 60%-84% of cases. Povidone iodine has shown similar results with a response achieved in 88% [15]. Nonetheless, it is not without dangers. Silver nitrate therapy in high concentrations may cause acute necrotizing urethritis with obstructive uropathy. Other more common side effects, including nausea, vomiting, flank pain, and haematuria, are usually transient. Not all patients respond well to sclerotherapy with 10%-40% of patients suffering a recurrence of chyluria [4]. In our case, sclerotherapy was unsuccessful. In a study following 72 patients with chyluria, Ohyama et al. found a 50% rate of spontaneous remission of chyluria. In most remission cases chyluria lasted for less than six months, although the duration time varied from three days to 20 years [16]. Other studies have also reported spontaneous remission in non-parasitic chyluria. The reason for spontaneous remission, however, remains unclear, and may be due to a collapse of the lymphatico-renal fistula or sclerosis of lymphatics caused by contrast agents, such as ethiodised oil-based contrast material [17]. When caused by filariasis, chyluria is treated with diethyl carbamazine. Alternative drugs include albendazole, ivermectin, and in some cases even doxycycline. Depending on the microfilaremic burden and in severe cases a febrile reaction may occur, therefore an antihistaminic agent is usually added to the therapy [14]. If all other treatment options fail to produce results, a pyelo-lymphatic disconnection procedure may be required [4].

## Conclusions

Repeated issues with the urinary system with regards to chyluria may present a difficult diagnostic and therapeutic dilemma. In our case report, we found CEUS and MR imaging techniques to complement each other greatly, with no additional exposure to radiation. CEUS retrograde pyelography provided decent visualization of the kidney’s hollow chamber and site of the leak, while MR imaging techniques gave more insight into the anatomy of the lymphatic system. Although contrast-enhanced MR lymphangiography in combination with T2 weighted MR did not reveal the location of the fistula in our case, the reviewed studies demonstrated that it is nevertheless the recommended option for visualizing fistulas in similar cases. Furthermore, we also discovered that the bubbles of the US contrast agent sulphur hexafluoride may temporarily close the communication between the lymphatic and urinary system.
